# “We Help Each Other Through It”: Community Support and Labor Experiences Among Brazilian Immigrants in Portugal

**DOI:** 10.3390/bs15091283

**Published:** 2025-09-19

**Authors:** Iara Teixeira, Patricia Silva, Felipe Alckmin-Carvalho, Guilherme Welter Wendt, Henrique Pereira

**Affiliations:** 1 School of Psychology, University of Minho, Campus de Gualtar, 4710-057 Braga, Portugal; iara.teixeira@ubi.pt; 2 Department of Psychology and Education, Faculty of Social and Human Sciences, University of Beira Interior, Pólo IV, 6200-209 Covilhã, Portugal; pg.silva@ubi.pt (P.S.); felipealckminc@gmail.com (F.A.-C.); 3 Research Center in Sports Sciences, Health Sciences and Human Development (CIDESD), 5001-801 Vila Real, Portugal; 4 School of Medicine, Western Paraná State University, Francisco Beltrão 85601, Brazil; guilherme.wendt@unioeste.br; 5 RISE-Health, Department of Psychology and Education, Faculty of Social and Human Sciences, University of Beira Interior, Pólo IV, 6200-209 Covilhã, Portugal

**Keywords:** Brazilian immigrants, labor precarity, xenophobia, mental health, community resilience, intersectionality, minority stress theory, coping

## Abstract

Over the last few years, the number of Brazilian immigrants living in Portugal has risen significantly, motivated by expectations of safety, prosperity, and professional success. However, the integration into the labor market frequently involves adversities such as professional devaluation, precarious working conditions, and experiences of social exclusion. This qualitative study aims to explore the work experiences of Brazilian immigrants in Portugal, with a special focus on how community support and collective resilience shape their ability to cope with adversity. Based on minority stress theory and intersectionality, we conducted 24 semi-structured interviews with Brazilian immigrants from diverse professional backgrounds. Thematic analysis revealed four main themes: (1) precarious integration into the labor market and underemployment, (2) experiences of xenophobia, racism, and discrimination, (3) mental health challenges and emotional exhaustion, and (4) community support and collective resilience. Participants emphasized the importance of informal solidarity networks to overcome institutional barriers and maintain emotional well-being. These results suggest that resilience is not only an individual resource, but a relational process rooted in everyday acts of care and connection. The study highlights the protective role of community in contexts of structural vulnerability and contributes to current discussions on migrant integration and well-being.

## 1. Introduction

The migration of Brazilian people to Portugal has risen over the last two decades, driven by historical, linguistic, sociopolitical, and economic factors. The shared colonial heritage, common language, and perception of Portugal as a “gateway to Europe” make the country a prime destination for those seeking better living conditions, security, and professional fulfilment (Azevedo et al., 2022). According to the latest “Foreigners in Portugal” report by the Agency for Immigration and Mobility—AMA (Lopes & Sousa, 2024), Brazilians are the most numerous foreign populations residing in the nation and account for 35.3% of legally resident immigrants. It is predominantly a young and economically active population, with approximately 80% between 25 and 44 years, having a fairly equal gender distribution (53% male, 47% female), thus resulting in a dynamic profile that contributes to the Portuguese workforce, to social security resource accumulation in Portugal, as well as to the rise in the country’s birth rate, which is significant due to the aging population (Lopes & Sousa, 2024).

Despite the significant presence of the Brazilian community in Portugal, many face obstacles to having their skills recognized in the country. Even when highly qualified, they often find themselves in low-paid jobs with limited protection, such as services, hospitality, and domestic work (Vidal-Coso & Vono de Vilhena, 2015). Even when they possess advanced educational qualifications, many immigrants face substantial barriers to accessing skilled positions (Maiztegui Oñate et al., 2023; Romens & Vianello, 2024). In addition, the perception that Portuguese employers value immigrants more for their willingness to work hard than for their formal skills reinforces a system of symbolic devaluation, especially for those who, alongside being immigrants, belong to other marginalized categories, such as women, the LGBTQIA+ population, and ethnic and racial minorities (Nascimento et al., 2023; L. B. Santos, 2013).

In this sense, although the migratory narrative surrounding Portugal still appeals to elements such as security, stability, and cultural affinity, the experiences of Brazilians in the country are mostly shaped by tensions between desire and frustration. As result of this situation, staying in Portugal does not always turn into a long-term plan. Data from AIMA (Lopes & Sousa, 2024) reveal that Brazilians accounted for 52% of notifications of voluntary abandonment of residence and 81% of beneficiaries of the Voluntary Return Support Program, indicating a mismatch between migration expectations and actual reception conditions.

This scenario becomes even more critical in the current political context. In June 2025, the Portuguese government presented a proposal for reforming migration policy. If approved, the measures include the requirement of prolonged legal residence for access to nationality, the prohibition of converting tourist visas into residence permits, limitations on family reunification only for highly qualified professionals, and the creation of the UNEF (National Unit for Foreigners and Borders), subordinate to the Public Security Police (Governo de Portugal, 2025). Although justified as rationalization mechanisms, these proposals come amid an escalation of the narrative of “uncontrolled immigration,” promoting the image of immigrants, including Brazilians, as subjects under surveillance, whose permanence would depend on constant demonstration of merit, productivity, and cultural conformity.

This type of discourse has direct repercussions on social and institutional reality. Also in June 2025, the European Commission against Racism and Intolerance (ECRI), linked to the Council of Europe, published a report warning of a significant increase in hate speech, both online and in the Portuguese community and political sphere. The document calls for more effective action against hate crimes and racial discrimination, highlighting structural flaws in the response of police forces, including in relation to extremist groups. In addition, ECRI criticizes the absence of a national integration plan and points to serious shortcomings in housing, health, and education policies aimed at migrant populations. Poor housing conditions, delays in regularization processes, and reports of institutional violence exacerbate the psychosocial vulnerability of these groups, revealing the multiple dimensions of exclusion experienced by immigrants in Portugal (Council of Europe, 2025).

These overlapping and multiple exclusions have a profound psychosocial cost for migrants and reinforce their vulnerability. Empirical research suggests that Brazilian immigrants who face labor precarity, discrimination, and institutional disregard reflect more severe levels of psychological distress, including anxiety, depression, and emotional exhaustion (Alarcão et al., 2023). For Brazilian women in particular, victimization by violence, invisibility socially, and institutional racism have been found to be linked with compromised self-reported health, heightened loneliness levels, and greater vulnerability to mental health illness (E. N. Oliveira et al., 2018). These findings are not merely the result of individual vulnerability, but the cumulative impact of structural stressors.

It is precisely in this scenario of institutional uncertainty, increased hostility, and policy restriction that the persistence of so many Brazilians becomes particularly striking. A study by Azevedo et al. (2022) shows that even when facing precarious working conditions and difficulties in obtaining professional recognition, many migrants are motivated by the perception that, despite the challenges, life in Portugal still represents an improvement compared to Brazil. However, this perception alone does not fully explain their ability to withstand the multiple vulnerabilities to which they are exposed.

Studies indicate that the support provided by fellow Brazilians plays a crucial role in sustaining migrants’ well-being in adverse environments. Through informal solidarity networks, such as friendship circles, digital communities, and mutual aid practices, Brazilian immigrants build emotional, informational, and material support systems that help them cope with exclusion, bureaucratic obstacles, and the psychological toll of migration (E. N. Oliveira & Neto, 2017; Padilla, 2008). These practices can be understood as forms of collective care, referring to everyday practices of support, solidarity, and mutual aid within migrant networks. Collective care has been described as a political and relational practice of sustaining life and well-being, particularly salient in times of crisis when institutional support is lacking (Chatzidakis et al., 2020). At the same time, these practices also illustrate processes of collective resilience, which denotes the broader relational capacity of communities to endure and transform adversity through cohesion, solidarity, and shared meaning-making. Research has shown that collective resilience is fostered by social cohesion and community-level coping strategies (Ludin et al., 2019; Liu et al., 2022), and that it is sustained by historical narratives of group endurance and resistance that nurture a sense of collective continuity and mobilization (Selvanathan et al., 2023; Vandrevala et al., 2024).

Rather than a collection of disconnected coping mechanisms, resilience in this case arises from social practices outside of everyday life. It is rooted in communal routines, mutual support, and the capacity of the community to generate space for shelter, meaning, and stability in the face of ongoing adversity. It is especially important because migration is typically an isolated experience, marked by the disintegration of known points of reference, social isolation, and affective exposure. That not only helps provide solutions to day-to-day issues, but also establish a sense of belonging, cultural continuity, and psychological comfort.

These patterns are also linked to Portugal’s colonial history with Brazil, which established enduring symbolic hierarchies that continue to shape labor market dynamics. Contemporary studies demonstrate that Brazilian migrants are often perceived through racialized and colonial stereotypes that position them as subordinate or less qualified workers, regardless of their formal education (Peralta et al., 2022). While Brazilian women may be especially targeted through gendered and sexualized forms of discrimination that construct them as “colonial bodies” (França & Prange de Oliveira, 2021), these dynamics extend more broadly to Brazilian men and other racialized migrants, who also face devaluation and exclusion in the workplace. Analyses of reciprocal migration flows further reveal how coloniality sustains the racialization of skills and contributes to labor market stratification across post-imperial contexts (Augusto et al., 2022). In addition, survey data confirm that racial and ethnic discrimination remains pervasive in Portugal, restricting opportunities for Black and non-white immigrants and reinforcing structural barriers to upward mobility (M. H. Santos et al., 2023). Taken together, these insights underscore how historical legacies of colonialism continue to shape symbolic hierarchies and sustain the devaluation of Brazilians in contemporary employment relations.

The present study is informed by three complementary perspectives. Minority stress theory (Meyer, 2003) highlights how stigmatization generates chronic stressors with negative consequences for mental health and well-being. Intersectionality (Crenshaw, 2013) underscores that these stressors are not experienced uniformly, but are compounded by the overlapping effects of race, gender, sexuality, and migration status, creating interdependent systems of disadvantage. Finally, the concept of collective resilience emphasizes that resilience is not merely an individual trait, but a relational process grounded in cohesion, solidarity, and collective meaning-making. Research has demonstrated that everyday practices of solidarity and mutual aid—often referred to as collective care—function as political and relational strategies to sustain life within marginalized communities (Chatzidakis et al., 2020). Beyond these practices, collective resilience refers to the broader capacity of communities to endure and transform adversity, and has been empirically associated with social cohesion, adaptive community responses, and the mobilization of historical narratives of endurance (Ludin et al., 2019; Liu et al., 2022; Selvanathan et al., 2023; Vandrevala et al., 2024). Together, these perspectives provide a lens to understand how Brazilian immigrants in Portugal navigate labor precarity, social exclusion, and psychosocial distress while simultaneously mobilizing networks of care and resilience.

Consequently, drawing on the lived experiences of Brazilian immigrants in Portugal, this research explores how migrants navigate labor precarity, perceived devaluation, and psychosocial strain, while simultaneously constructing networks of care, solidarity, and collective resilience. By bringing together these experiences of hardship and support, this study adds to ongoing debates on migration and labor, illustrating the need for policies that consider the realities lived by migrant populations. The purpose of this paper is not only to document Brazilian immigrants’ labor experiences but also to clarify how these experiences illustrate and expand the concept of collective resilience. By situating everyday practices of solidarity within the frameworks of minority stress and intersectionality, the study highlights the theoretical relevance of collective resilience as a lens for understanding migrant coping and adaptation.

Based on the theoretical framework of minority stress theory, intersectionality, and collective resilience, this study was guided by the following research questions:

1. How do Brazilian immigrants in Portugal experience labor precarity and professional devaluation, and how do these conditions shape their everyday vulnerabilities?
2. In what ways do Brazilian immigrants report experiences of xenophobia, racism, and other forms of discrimination in the workplace, and how are these stressors compounded by intersecting identities?
3. How do experiences of precarity and discrimination affect immigrants’ psychosocial well-being, particularly in terms of stress and minority stress processes?
4. How do Brazilian immigrants mobilize practices of collective care, and how do these practices contribute to broader processes of collective resilience in navigating labor and social adversity?

## 2. Materials and Methods

This is a qualitative, exploratory, and descriptive-analytical study that aims to understand Brazilian immigrants’ experiences in the world of work in Portugal. A qualitative approach was chosen due to the subjective and contextual nature of the experiences investigated, as well as the intent to capture the meanings attributed by the participants to their own trajectories. Our research was funded by RESTART, a program of the Foundation for Science and Technology, Portugal (grant number: 2023.00018.RESTART).

### 2.1. Participants and Selection Criteria

This research gathered participants that are over 18 years of age, Brazilians, residents in Portugal, and have had experience in the formal or informal labor market. The recruitment process involved a combination of collecting participant contact information from referrals from the first author’s network. From the first participants, during the interview they were asked to indicate other participants who could take part, resulting in snowball sampling (Browne, 2005). The number of participants was defined based on the principle of theoretical saturation, which occurs when new data no longer make relevant contributions to the analytical categories (Guest et al., 2006).

### 2.2. Data Collection

Once potential participants were identified, an initial contact was made via email or phone to introduce the study and its objectives, and to explain how the interview would be conducted. Participants scheduled an interview time at their convenience using the Teams platform. The interviews were recorded with the participant’s consent and then fully transcribed for analysis. The length of the interviews was not pre-established and interviews lasted an average of one hour. Participants did not receive financial incentive for participating in the study. The interviews were conducted based on a semi-structured set of questions, using a script that covered topics such as daily life at work, relationships with colleagues and superiors, perceived discrimination, and well-being strategies. Data collection was conducted during the first semester of 2025. Table 1 shows the list of questions applied.

In addition, to characterize the participants, the following sociodemographic data were collected: age, sexual orientation, marital status, formal education, economic status (self-classified as low, low-middle, middle, middle-high or high), occupation, and time in employment.

All interviews were conducted and analyzed in Portuguese. For publication, the excerpts presented in this article were translated into English by the first author, who is bilingual, and subsequently reviewed by the co-authors to ensure accuracy and fidelity to the participants’ original meanings.

### 2.3. Analysis Procedures

The emerging content was analyzed using the thematic analysis technique (Braun & Clarke, 2021), with the support of MAXQDA 24 (VERBI Software, 2025), which helped with the coding, organization, and visualization of the data. The software was used exclusively as a technical tool for structuring the material, whereas theoretical interpretation was carried out in a subsequent phase. The analytical framework was grounded in the theories of minority stress (Meyer, 2003) and intersectionality (Crenshaw, 2013), which were mobilized during the interpretative stage to guide how broader categories were understood and analyzed. Additional concepts, such as collective care and collective resilience, emerged inductively from participants’ narratives and were later connected to the literature to deepen the interpretation. The analytical process considered all the COREQ criteria (Tong et al., 2007), ensuring transparency and rigor in qualitative reporting, and followed the following steps: 1. Floating reading of the transcripts; 2. Initial coding of speech segments based on the themes in the script and the emerging themes; 3. Grouping codes into broader thematic categories; and 4. Interpretation of the categories in the light of the study’s theoretical assumptions, linking participants’ accounts to processes of minority stress and intersectional exclusion.

The analysis sought to preserve the complexity and uniqueness of the narratives, without diluting the meanings in excessively abstract categories. Given her shared background with participants, the first author acknowledges the potential for interpretative bias in the analysis. To mitigate this, coding categories and emerging themes were systematically discussed and revised in collaboration with the co-authors, allowing for critical reflection and ensuring greater analytical rigor.

### 2.4. Ethical Considerations

All ethical policies were thoroughly implemented, and approval was sought from the Ethics Committee of the University of Beira Interior, Covilhã, Portugal (Protocol No. CE-UBI-Pj-2024-022-ID2212, 16 April 2024). After being fully informed about the study’s purpose, procedures, potential risks, and benefits, participants provided informed consent before the start of the study. All aspects of confidentiality and privacy were strictly maintained. All data, including audio files and transcripts, were securely stored in encrypted, password-protected folders on the University of Beira Interior’s institutional servers, with access restricted to the research team. Lastly, the study respected transparency for reporting results, consistent with the Declaration of Helsinki (World Medical Association, 2013) ethical guidelines.

## 3. Results

This study included 24 Brazilian immigrants with work experience in Portugal (mean age 31.67; SD 5.34). The sample was quite diverse, covering participants from different sectors of work. We had participants who work in technology (e.g., programmer, backend developer), health (e.g., medical translator), education (e.g., assistant professor), services (e.g., café attendant, waitress), industry (e.g., production assistant and operator, textile reviewer), and communication (e.g., advertising), as well as informal and/or self-employed occupations. Detailed information about sociodemographic information can be found in the Supplementary Material.

The thematic analysis of the interviews revealed four primary themes, which were recurring patterns in the participants’ narratives: (1) Precarious Job Integration and Professional Disqualification, (2) Discrimination, Xenophobia, and Racism in the Workplace, (3) Psychosocial Impacts and Mental Health; and (4) Coping Strategies and Support. Each theme is described in the following subsections.

### 3.1. Precarious Job Integration and Professional Disqualification

Although 76% of our participants have education beyond secondary school, the interviews revealed that most of them, upon arriving in Portugal, end up facing a mismatch between their professional qualifications and their occupation. These patterns are not merely transitional; they reveal institutional constraints, such as the dependence on employment contracts for legal status and the urgency of immediate income, which channel migrants into survival jobs. Analytically, these dynamics can be understood as distal minority stressors, in which institutional barriers and structural exclusion produce chronic vulnerability and long-term psychosocial strain (Meyer, 2003). It is possible to identify in their statements a feeling of frustration because they believed that having a formal education would enable them to find a job in their field.

“I had just graduated thinking that getting a job [in my professional area] would be the easiest thing in the world. […] That was the initial plan, to work according to my training. […] It wasn’t like that. It was a whole process to get documentation and then you had to have a contract. […] I worked at a kiosk on the beach, an ice cream stand, and I also worked at a Japanese restaurant. And so, these were experiences from hell.”

—P8

This account illustrates the dissonance between expectations and reality, as well as early status loss, which are typical entry points into minority stress processes where structural barriers are internalized as frustration and diminished self-worth. The need to secure income swiftly often leads to the acceptance of precarious employment, frequently without formal contracts, long working hours, a single day off per week, or even two jobs to cover expenses.

“I exchanged housing for cleaning the office [of the company that provided me accommodation], which was huge. I worked eight hours straight and then went to clean, but other than that, I received my salary. At that time I couldn’t get a contract, […] the employers did everything within the law and ended up being afraid of inspections. […] Then I left there, went to work in a factory, worked as a cleaner, worked as a caregiver for the elderly, and later with Uber Drive and Uber Eats, which I still do from time to time [alongside my current job].”

—P20

This testimony illustrates how institutional vulnerability and economic coercion converge, intensifying exposure to both distal stressors (informality and unstable contracts) and proximal ones (daily uncertainty and insecurity). Often, these jobs are seen as a temporary stage, a necessary step to adapt while waiting for an opportunity to work in a position more suited to their qualifications. However, this desire is sometimes undervalued by employers. Blocked mobility also appears when aspirations are dismissed or ridiculed by employers, reinforcing symbolic devaluation.

“In the interview, I told [the employer] clearly: I’m accepting this position [outside my area of training], but I’m trained as an administrative assistant, and that’s the path I want to follow. So, whenever a position opens up in that area here, I’m ready to take it. He just looked at me and laughed.”

—P13

Such reactions function as status cues that migrants are “out of place,” intensifying minority stress and prompting anticipatory self-exclusion from opportunities for professional progression. A minority of participants subsequently secured a position in their desired field of work (n = 11), as is the case of Participant 3, who is now a university professor. However, memories of their experiences in hostile work environments remain:

“Before I started teaching, I worked in a call center, in restaurants, and in the kitchen of a care home [for people with disabilities]. The worst environment I experienced was in restaurants, mainly because of the rude way people treated me. But after I started teaching, I am grateful that at least I haven’t directly experienced that kind of mistreatment.”

—P3

Even upward movements did not erase the stress legacy of earlier experiences of devaluation. For most, the transition to jobs that match their qualifications remains limited. There is a feeling that there are structural barriers imposed on immigrants in the labor market, with discouragement emerging in their statements after countless unanswered attempts:

“When I tried to change job positions within the company where I work—for example, into economics or administrative roles—I just couldn’t get in. It’s much more closed off to [Brazilian immigrants]. Imagine sending your résumé already knowing it’s going to be ignored. That’s basically the reality.”

—P9

This statement reflects anticipated discrimination and learned futility, proximal minority stress responses that erode motivation and well-being. Even though they are employed, many participants reported not feeling valued or recognized in their workplaces. This devaluation appears in different forms: wage differences, micromanagement, exclusion from opportunities for advancement, or simply being treated as “replaceable labor.”

“You do the same work as [Portuguese colleagues], but because you’re an immigrant, it doesn’t have the same value. You don’t get paid the same.”

—P20

“I can never plan my day, so I have a series of activities that I have to do. I set aside time in my schedule, and even then, [managers] don’t respect it. It makes me anxious and a little demotivated. I haven’t found opportunities for career progression in any company I’ve worked for here.”

—P21

These quotes reveal that the experience of devaluation is subjective and structural, affecting both those in jobs below their qualifications and those who work formally in their field. Lack of recognition appears to be yet another obstacle to the full integration of immigrants into the Portuguese labor market. From a theoretical perspective, these dynamics reflect the accumulation of distal and proximal minority stressors: institutional barriers and de-skilling on the one hand, and everyday practices of devaluation on the other. The result is a cycle of frustration, demotivation, and anxiety that undermines well-being while normalizing inequality as part of everyday working life. This normalization of exclusion not only perpetuates feelings of invisibility but also prepares the ground for the xenophobia, racism, and other forms of discrimination that Brazilian immigrants report in the Portuguese workplace, which we examine in the following section.

These accounts reveal not only structural labor market segmentation but also the psychosocial toll of minority stress, as experiences of professional downgrading and lack of recognition translate into both distal and proximal stressors that undermine migrants’ well-being.

### 3.2. Discrimination, Xenophobia, and Racism in the Workplace

Discrimination was reported in various ways. These experiences were described as both explicit xenophobia and more subtle and normalized practices, such as jokes, exclusion, and stigma associated with being Brazilian. These accounts align with the framework of minority stress, where external prejudice and stigma act as distal stressors that undermine well-being (Meyer, 2003). In some cases, these episodes were exacerbated by overlapping social markers, such as gender, race, or sexual orientation. This points to intersectional dynamics (Crenshaw, 2013), where multiple stigmas compound disadvantage. Several participants reported feeling that they were treated differently based solely on their nationality. Even when performing the same duties, they perceived a difference in treatment, often with a tone of inferiority.

“When I arrived in Portugal, I faced a lot of discrimination for being Brazilian—mostly in the way I was treated, like being given more work or harder tasks [by supervisors] than others. I clearly saw how differently I was treated compared to Portuguese coworkers.

—P18

This statement illustrates how discrimination becomes normalized in daily tasks, reinforcing symbolic devaluation and fueling feelings of frustration consistent with minority stress processes. Some reports highlight situations of direct rejection and explicit hostility on the part of customers or colleagues.

“For me, it was the worst moment: being mistreated. I felt humiliated. You’re there, working, trying to do your best to serve [a customer], and they say: ‘Oh, but you’re Brazilian… I don’t like you.’ And it wasn’t said in a calm tone, it was harsh, ironic, with a look of disgust. With an air of superiority, of someone who thinks they are better. I think it was one of the worst moments I ever had there.”

—P2

This account represents an acute minority stressor: an explicit act of xenophobia that produces humiliation and long-lasting psychological costs. Xenophobia does not only manifest itself as direct insults; it is incorporated into forms of disguised paternalism, where Brazilian workers are perceived as people who do not understand instructions or recommendations, and need to be constantly corrected, even when they demonstrate competence, or people who do not respect the rules, and who need to be constantly supervised.

“There’s something curious about Portuguese people: [colleagues and supervisors] often think we know less than they do, so they feel they need to explain things at length that we already… no, no, I already understood, I already understood, but they’re there trying to explain, explain, explain, and you feel a certain… at that moment I feel a little… it’s a bit irritating, like… they think we don’t understand, like we don’t have the intellectual capacity to understand what they’re talking about.”

—P14

This illustrates subtle but chronic forms of discrimination, often experienced as microaggressions. These reinforce stereotypes of incompetence and erode professional identity over time. In addition to nationality, other social markers intensify experiences of discrimination. During the interviews, there was a recurring theme of intersectional oppression, with participants being simultaneously marked as foreigners, women, Black people, or LGBTQIA+ individuals.

“Just the fact that you are a woman and Brazilian means you are already viewed negatively, right? There is a whole urban legend [stereotype that Brazilian women are overly sexual or associated with prostitution] in Portugal. So just being a woman seems like a big threat. I always knew I was a Black woman and everything, but in Europe it became so obvious, it became so clear in my head, it was like every day someone made a point of rubbing it in my face in such a negative way that I was like, my God, so there you have it, racism, that’s really racism, and it’s not veiled racism, it’s overt racism, where people really aren’t ashamed [of being openly racist]

—P8

This testimony shows how intersectionality magnifies exclusion: nationality, gender, and race overlap to produce cumulative forms of stress and marginalization. Furthermore, even when victims choose to report racism, xenophobia, or moral harassment, testimonies point to an organizational culture of impunity. This ultimately discourages victims of labor or human rights violations from reporting their situations. Participants also reported fear of reprisals from their employers, especially in small towns, with some cases involving explicit threats. Finally, some participants mentioned that most organizations do not offer effective responses to complaints. and in some cases, the victim ends up being punished.

“And [a colleague] filed several complaints. And what did they do? Nothing. What [the harasser] did was call the manager in for a chat. Maybe gave her a little scolding—or not even that. They probably just had a nice coffee together. Because in the end, [the colleague who reported it] was the one who got fired.”

—P4

This example highlights institutional betrayal, where attempts to denounce discrimination result in further victimization. Such dynamics intensify minority stress and reinforce migrants’ mistrust of organizational protections. Such experiences reflect the intersectional nature of minority stress, where nationality, gender, race, and sexuality interact to produce compounded exclusion and intensify psychosocial vulnerability. Participants who were exposed to a variety of adverse situations on a chronic basis experienced significant impact on their emotional and psychological health, the theme presented in the next thematic axis.

### 3.3. Psychosocial Impacts and Mental Health

The set of difficulties reported by participants significantly impacts their emotional well-being, quality of life and self-esteem, as well as being risk factors for the emergence or worsening of mental health problems The reports reveal experiences marked by anxiety, stress, exhaustion, constant fear of losing their jobs, and feelings of loneliness and existential emptiness, especially among those without a local social or institutional support network. These consequences align with minority stress theory (Meyer, 2003), since repeated exposure to exclusion and devaluation translates into chronic strain on mental health.

“I had severe anxiety attacks, I kept asking myself, Why the hell am I here? Why did I come? Do I really need this? What’s the point?’ then, like, I started to get into a loop, like, really questioning myself. […] situations like that [referring to episodes of discrimination] were what affected me, my psyche in a way I didn’t even know was possible, where you feel like a piece of shit, you know, you feel kind of broken, right? They [Portuguese colleagues and employers] see me as nothing here, that’s how I’m seen, that’s how I’m perceived, no matter how much I talk, it’s not going to be heard.”

—P8

This account illustrates the cumulative toll of minority stress, where persistent devaluation and discrimination translate into acute anxiety and identity erosion. The initial period of life in Portugal, marked by the process of adaptation and separation from previous connections, was particularly challenging for some participants, who reported symptoms consistent with depression, anxiety disorders, adjustment disorder, or burnout, with the absence of family or close friends intensifying their psychological distress.

“It was during my first period in Portugal, all that depression that migrants experience, whether you want to or not, even though you know you’re studying, you’re doing other things, you already have that cultural shock. And it’s also very lonely, immigration is always very lonely, you leave a lot behind.”

—P22

This testimony points to adjustment stress compounded by social isolation, a proximal stressor that heightens vulnerability in the absence of local support. In addition to emotional distress, the reports reveal a recurring fear of losing one’s job, which leads to remaining in unhealthy environments, even in extreme situations. The following is an account of the loss of pregnancy while at work and the decision to continue working for fear of being fired.

“During that period, I found out I was pregnant, and I continued working in silence, mainly because I was afraid of being fired. […] I lost the baby, I spent a whole month bleeding and with a fever, and I couldn’t stop working. […] I called her [my supervisor] and said, “Look, I’ve lost a lot of blood, I’m covered in blood and alone.’ Then she was extremely rude and asked, ‘What do you want me to do? There’s no one to replace you. I’m here at home, I can’t leave, what do you want me to do? Do you want to go home?’ Then I said, ‘No, no, it’s okay, I’ll figure something out,’ and I stayed.”

—P1

This case exemplifies institutional vulnerability and self-silencing driven by job insecurity—core features of minority stress that can have severe psychosomatic consequences. Psychological suffering is also exacerbated by the perceived lack of awareness or respect shown by managers towards the emotional needs of workers. Work is experienced as a place of oppression and silence.

“Because bosses only care about profits, right? They don’t care about the mental health of the people who work for them, they just want you to work to make more money for them, they don’t care about the environment or the atmosphere. That’s why they hire so many foreigners [immigrants], because foreigners have to pay rent. To produce capital for those who are already rich.”

—P8

Narratives of instrumentalization and disregard for well-being contribute to perceived injustice and demoralization, reinforcing proximal stress responses (e.g., anxiety, hopelessness). Many participants associate their emotional deterioration with authoritarian and disrespectful forms of leadership.

“It’s a feeling I get in Portuguese companies. At the leadership level, they don’t really know how to lead. It’s more like beating a dead horse [constantly pushing people beyond their limits]. It’s very aggressive, let’s say.”

—P21

Perceptions of authoritarian leadership operate as everyday micro-stressors that sustain hypervigilance and emotional exhaustion. Faced with these contexts of suffering, emotional overload, and hostile work relationships, some participants described ways of resisting or protecting themselves, both through social networks and emotional bonds, and through seeking professional mental health support.

Although psychosocial effects were recurrent across the sample, their intensity and form varied according to sociodemographic profiles (see Supplementary Table S1). Racialized women reported particularly intense intersectional discrimination (e.g., P8, P24). Participants with lower education or employed in low-protection sectors (e.g., P10, P22) faced more acute contractual exploitation and instability, whereas those with higher education but relegated to downgraded jobs (e.g., P30) expressed strong frustration and devaluation. This heterogeneity illustrates how gender, race/color, education, and occupation intersect in the production of minority stress and help to contextualize differences in the psychosocial impacts described.

The psychological suffering described here is consistent with minority stress processes, where structural and interpersonal discrimination generate chronic emotional strain and health risks. In line with our broader argument, these coping efforts anticipate the role of community-based strategies discussed next. These coping strategies and subjective care are explored in the following section.

### 3.4. Community Support and Collective Resilience

As participants navigated various forms of precarity, exclusion, and emotional strain, a recurring element in their narratives was the importance of community-based support. For many, the solidarity of other Brazilians functioned as the first and sometimes only line of protection against isolation and instability. These networks emerged not through formal structures, but through everyday relationships built on shared experience, nationality, and empathy. This illustrates what we conceptualize as collective care, the relational practices that immediately sustain well-being in contexts of structural exclusion.

Upon arrival in Portugal, the majority of participants had already ended up in precarious and exposed situations, without institutional support or access to services. In these contexts, informal assistance from other Brazilians was significant. Participants described being welcomed in friends’ or acquaintances’ homes, accompanied through bureaucratic processes, and aided with work or housing.

“My friend [from Brazil] who was already living in Portugal helped me with everything. I came and stayed at her house for a while, until I got settled.”

—P1

This type of support exemplifies collective care as a substitute for absent institutional protection.

“An acquaintance referred me for a caregiver position. I had no experience, but she spoke for me [personally recommended me to the employer]. I got the job because of her.”

—P10

Here, solidarity directly translated into access to employment, showing how care networks mitigate barriers to labor market entry. This type of informal support tended to make up for institutional breakdown. For others, it was more than a survival tool, but a way of maintaining a human sense of dignity during adaptation. These connections evolved into deeper emotional bonds, and some participants mentioned friends who “became family,” offering companionship and emotional holding in times of crisis.

“I have a friend here who has become like family to me. When I go through something difficult, she is the one who listens to me. Without her support, I think I would have gone back [to Brazil].”

—P3

This statement highlights how solidarity moves beyond instrumental support, becoming a source of belonging and continuity, which is central to collective resilience. These support networks extended into online realms. Participants explained how online platforms and social media groups served as meeting points where they would assemble with other migrants and be able to access important information.

“When I arrived, I hardly knew anyone. Then a Facebook group connected me with a Brazilian woman [a stranger at first] who took me into her home until I found a room to rent.”

—P3

Digital spaces thus emerged as extensions of collective care, broadening access to protection and support. Workplaces, to the extent that they were fields of conflict or discrimination, were also sites where Brazilians found solidarity in solid forms. Shared struggles fostered empathy and mutual understanding.

“We help each other through it, especially among Brazilians. Some days at work, we just need to look at each other to know that someone isn’t feeling well.”

—P3

This account reveals how solidarity can be silently enacted even in hostile environments, reinforcing collective resilience against everyday discrimination. These findings suggest that, whereas some participants identify one-on-one strategies to deal with emotional distress, such as therapy or setting boundaries, it is the collective dimension of care that is most evident. Instances of solidarity, such as offering accommodation to live, exchanging employment tips, or simply listening, weave a network of everyday protection that facilitates wellness in the absence of official care. In theoretical terms, these practices are not only forms of collective care but also illustrate collective resilience, understood as the relational capacity of communities to endure and transform adversity through cohesion, solidarity, and continuity (Selvanathan et al., 2023). These narratives illustrate how everyday practices of solidarity evolve from forms of collective care into collective resilience, creating a relational capacity to endure and transform adversity in the absence of institutional protection. For Brazilian immigrants facing systemic marginalization and exposure, community emerged in our participants’ accounts as a central site of resilience. It is within these shared experiences that they create recognition, continuity, and the resilience to navigate the life of migration. In operational terms, our analysis coded these immediate practices as forms of collective care and interpreted their accumulation and continuity as collective resilience, understood as a relational capacity sustained through ongoing solidarity and recognition.

## 4. Discussion

This study aimed to examine how Brazilian immigrants navigate the labor system in Portugal, including experiences of job insecurity, discrimination, and their psychosocial consequences, as well as how they mobilize practices of collective care that contribute to broader processes of collective resilience. The findings show that participants frequently described conditions of underemployment, symbolic devaluation, and workplace discrimination, reinforcing the persistence of structural barriers faced by migrant workers. These stressors were reported as having significant psychosocial costs, including anxiety, loneliness, and emotional exhaustion, which align with minority stress processes. At the same time, participants emphasized the importance of networks of solidarity and support, ranging from emotional and informational help to material assistance. We interpret these everyday practices as forms of collective care, which in turn foster collective resilience, understood as the emergent capacity of communities to endure, adapt, and transform adversity through cohesion and shared identity (Ludin et al., 2019; Liu et al., 2022; Selvanathan et al., 2023; Vandrevala et al., 2024).

### 4.1. Labor Experiences and Impacts

In Portugal, precarity in the working conditions, low wages, and disregard for the country’s labor laws has become widespread, affecting even native workers with different levels of education (Carvalho, 2014). However, studies such as those by C. R. Oliveira and Fonseca (2013) and, more recently, by Azevedo et al. (2022), point out that even though Portuguese people themselves face job insecurity, immigrants face more barriers and are more likely to work in jobs below their qualifications.

The literature on migrant labor in Europe reports the consistent pattern of systematic exclusion faced by professionals from the Global South. They can be highly qualified, but they are usually placed in low-income streams and prevented from professional progression. This incongruity is not a coincidence; it betrays a larger system shaped by colonial pasts and racialized labor hierarchies, in which Latin American migrants are supposed to naturally fit into subaltern or care work, irrespective of their qualifications (Purkayastha et al., 2023). All these trends are supported by the non-recognition of degrees acquired abroad and administrative barriers which devalue knowledge from the Global South (Pecoraro & Wanner, 2019), and symbolic stigmas, such as discrimination based on accent, that render Brazilian immigrants’ subordination in the labor hierarchy the norm (Śliwa et al., 2023).

Although this study focuses specifically on Brazilian immigrants in Portugal, many of the challenges described here resonate with the experiences of other migrant populations across Europe. In Italy, for instance, foreign workers face high levels of over-qualification, with 46.9% of EU-born migrants and 67.1% of non-EU migrants working below their skill level, compared to 19.1% of Italians (International Labour Organization, 2024). In Germany, economic immigrants and refugees commonly experience “skill downgrading,” occupying positions beneath their qualifications and receiving lower wage returns compared with natives with similar skills (Brell et al., 2021). More broadly, across the EU, migrants are disproportionately over-represented in essential sectors such as health, long-term care, food supply, and transport, often under insecure contracts, which reflect structural labor market segmentation rather than individual preference (Eurac Research, 2024). These parallels indicate that the barriers identified in this study are not isolated to Brazilians in Portugal but are part of systemic patterns within European labor and migration regimes, highlighting the importance of further comparative research.

Precarity worsens when intersectionality is considered. Research indicates that not all migrants are excluded to the same extent and precarity and discrimination are not distributed homogeneously. It varies with skin color, gender, sexual orientation, and social economic status (Intungane et al., 2024). In our study, racialized women, for example, reported explicit racism and stigmatization combined with gendered stereotypes, amplifying workplace vulnerability. Workers with lower education or in low-protection sectors experienced more acute contractual exploitation and insecurity, while highly educated individuals placed in downgraded jobs expressed frustration and feelings of devaluation. This intersectional reading highlights that minority stress is produced not only by immigrant status but through the entanglement of multiple social markers that structure labor market dynamics and mental health experiences.

In fact, Andrade (2023) describes how migrant Latin women are subjected to intersecting symbolically and materially the mechanisms of violence, such as denial of professional recognition and normalization of precarity even when they hold formal qualifications, a testimony to the structural nature of devaluing their knowledge and path. Even among those who find work that matches their qualifications, there is a persistent feeling of being undervalued. Practices such as micromanagement, excessive workloads, and lack of recognition are often associated with being a foreigner, fueling the idea that, regardless of performance, these professionals occupy a secondary social position (Ingwersen & Thomsen, 2024).

The participants also demonstrate how working conditions affect mental well-being, manifesting in symptoms such as anxiety, depression, and burnout. These findings are consistent with the systematic review by Ornek et al. (2022), which confirms the link between precarious employment among immigrants and mental health problems, particularly when combined with factors such as insecurity, work overload, and symbolic discrimination. The study further highlights institutional vulnerability as a key determinant of poorer mental health outcomes, since dependence on employment contracts for legal status fosters a profound sense of insecurity and constant vigilance, ultimately trapping immigrants in unstable jobs (Ornek et al., 2022).

Further qualitative evidence supports the observation that immigrant workers are susceptible to chronic psychological stress in poor working conditions induced by exploitative labor conditions, inadequate interpersonal treatment, and structural barriers to more decent work (Shankar et al., 2024). For racialized migrants or language problems, this stress is supplemented by negative social perceptions and the absence of autonomy, as well as disempowerment within the workplace. Furthermore, legal ambiguity, like dependency on labor contracts for residential status, continues to be a cause of pervasive fear and worry, amplifying emotional vulnerability (Horner & Woolford, 2023). Inaction or ineffectiveness of institutions also brings mental burden in that migrants lack access or have only limited access to the required support systems or cultural-sensitive care, inducing abandonment and loneliness feelings (Bailur et al., 2023).

### 4.2. Collective Care and Collective Resilience

Despite the adverse conditions set forth, this study also revealed how collective care and solidarity strategies function as central determinants of resilience for Brazilian migrants. Where institutional protection is absent or ineffective, the group becomes the foremost source of resources. Participants frequently recounted that they received shelter, job referrals, and moral support from fellow Brazilians, especially during moments of vulnerability and ambiguity. While participants often described everyday acts of support such as sharing housing, exchanging information about jobs, or providing emotional assistance, these practices can be best understood as collective care, that is, the immediate and relational strategies through which communities sustain one another in contexts of precarity and exclusion (Chatzidakis et al., 2020).

Beyond these concrete acts, however, the findings suggest that such practices also generate a broader relational capacity, which we conceptualize as collective resilience. This capacity does not reside in isolated individuals but emerges from the cohesion, solidarity, and continuity of the community, enabling migrants to endure structural vulnerabilities and to create meaning and belonging despite adversity (Ludin et al., 2019; Liu et al., 2022; Selvanathan et al., 2023; Vandrevala et al., 2024). By distinguishing between collective care and collective resilience, our analysis highlights that what may initially appear as fragmented or informal support systems are in fact constitutive of a collective capacity that sustains well-being and fosters possibilities for resistance and transformation.

This community-based resilience is in accordance with recent findings that demonstrate that social support is one of the most valid predictors of better mental health outcomes among immigrants. For example, a large study in Spain found that support from friends and fellow ethnics significantly improved migrants’ psychological well-being through increased feelings of belonging and contentment with life (Hombrados-Mendieta et al., 2013). Also, a Canadian population study attested to that insufficient social support was found to have a greater predisposition of mental health disorders among immigrants, whereas individuals with high social networks—particularly in long-term immigrants—were more protected against depression and anxiety (Puyat, 2013).

These findings corroborate what our respondents explained explicitly in their narratives: networks of friends, coworkers, and acquaintances were an essential element helping them navigate through bureaucratic hurdles, workplace mistreatment, and emotional crises. For many, community was not a passive backdrop, but an active presence that alleviated the burden of exclusion, creating a space where they might be observed, heard, and protected. The findings also resonate with research showing that historical collective resilience strengthens a sense of collective continuity: the perceived connection between past, present, and future struggles, which in turn sustains coping and mobilization in the face of ongoing adversity (Selvanathan et al., 2023).

This buffering effect of social support has also been shown to interact with the resilience traits of individuals, including self-efficacy and emotional control, to forecast positive outcomes for immigrants. García-Cid et al. (2017) found that both individual and collective resilience factors, including peer and family support, significantly influenced the emotional well-being of Latin American immigrants in Spain.

While individual coping mechanisms like therapy or setting emotional boundaries were occasionally mentioned in our study, the most robust and most common protective factor was the presence of other Brazilians as sources of solidarity. These findings underscore that in unsafe and frequently isolated migration environments, resilience is not merely an individual skill but also relational and community based. These everyday gestures of care among migrants—giving shelter, listening to a crisis-ridden friend, sharing job tips—constitute the basis through which survival and dignity psychologically are facilitated.

### 4.3. Implications

The implications of this study are multiple. In the field of public policy, it is essential to review the mechanisms for recognizing foreign academic qualifications and the criteria for migration regularization, to guarantee immigrants decent working conditions in accordance with Portuguese labor legislation. Moreover, there is an urgent need to implement effective policies to combat xenophobia, institutional racism, and the intersecting forms of discrimination experienced by Brazilians—especially women, Black people, and LGBTQIA+ individuals—in the workplace, including complaint channels that ensure protection for victims, as also highlighted by the European Commission against Racism and Intolerance (Council of Europe, 2025).

Given the political reality of immigration in Portugal today, this study is particularly relevant. The government of Portugal has been criticized by EU institutions and human rights organizations for its weak and slow institutional response to the increasing influx of immigrants. Criticism has been made against the legalization process, the acquisition of nationality, and access to public integration policy (Reis, 2023). Given this situation, the support networks that are formed among immigrants are even more essential.

By highlighting the precarious conditions experienced by Brazilians in Portugal, this research illustrates the gap between mobility ideals and the exclusionary realities of the host country. Such conditions only get worse with policies that expand barriers, withhold rights, and have no regard for immigrant communities’ social and economic contributions. It should be noted that, according to the latest Immigrant Integration Indicators report (Reis, 2023), the foreign population has contributed significantly to the sustainability of the social security system in Portugal. In 2022, immigrants represented 13.5% of total contributions, generating around 1.861 billion euros and receiving only 256.8 million in benefits, resulting in a net contribution of 1.604 billion euros. Brazilians accounted for 40.5% of this total, contributing more than 668 million euros. These figures challenge the narratives that associate immigration with the collapse of public services, highlighting instead the essential role that immigrants play in financing the country’s social protection system.

Clinical and institutional psychological practice points to the importance of a critical social psychology that recognizes the role of coloniality, xenophobia, and racism in the production of psychological suffering. Psychologists working with migrant groups must be prepared for practice sensitive to the intersectionalities that frame their lives, knowing that suffering is not individual but socially and historically situated.

Within academic discourse, this study illustrates the relevance of intersectional and psychosocial approaches to the understanding of migration and expands the focus to the integral role of solidarity networks. In its demonstration of how social support operates as a protection, a process of recognition, and an act of resistance, this study provides empirical support for perspectives that conceptualize resilience not simply as individual capacity but as collective power mobilized under duress.

By distinguishing between collective care and collective resilience, this study offers empirical insights that enrich ongoing discussions in the literature on migrant resilience. Recent scholarship has emphasized the need to theorize resilience not as an individual trait but as a relational and collective process (Liu et al., 2022; Selvanathan et al., 2023; Vandrevala et al., 2024). Our findings support this perspective by demonstrating that resilience among Brazilian immigrants is grounded in solidarity networks, shared histories of exclusion, and mutual support, highlighting its transformative potential in contexts of structural precarity.

By foregrounding the concept of collective resilience, this study demonstrates that the experiences of Brazilian immigrants in Portugal cannot be understood solely through the lens of precarity, discrimination, and psychosocial vulnerability. While these stressors remain pervasive, our findings show that migrants simultaneously mobilize relational resources that extend beyond individual coping. Through practices of collective care—material, emotional, and informational—communities generate a broader capacity to endure and transform adversity, fostering belonging, continuity, and resistance. This conceptualization of resilience as collective rather than individual highlights the importance of attending to relational and community-based strategies in migration research. It also underscores the need for policies that not only address structural barriers in the labor market but also recognize and strengthen the communal resources through which migrants sustain well-being and negotiate social inclusion.

### 4.4. Limitations and Future Directions

Although we met the objectives of the present research, we acknowledge some limitations. The sample, while diverse, was primarily recruited through snowball sampling, which may have limited the reach to more socially isolated groups. In addition, we did not collect standardized information on immigration status (e.g., residence permit) or race/color. Mention of these markers only occurred spontaneously within participants’ narratives, and while some racialized women explicitly discussed experiences of racism and stigmatization combined with gendered stereotypes, increasing work vulnerability, these data cannot sustain systematic subgroup comparison. Moreover, the study centered on work relations and did not study closely access to public services, housing, and education, which are equally essential to migratory experience.

Future studies should explore longitudinal or panel designs and mixed methods that would be valuable in tracing how minority stress and collective resilience develop at different phases of settlement and in different areas and economic sectors. Quantitative methods could supply estimates of solidarity networks’ contribution to mental health and labor outcomes, complementing this study’s qualitative depth. Comparative research with other immigrant groups in Portugal and across Europe would allow for both the assessment of transferability and contextual differences, particularly in terms of racialized and legal statuses. Further research could also examine intersectional differences by gender, race, sexuality, class, and legal status through targeted sampling and comparative sub-analyses. In addition, closer, more focused studies of specific models of community support, e.g., peer mentoring, mutual aid groups, or community centers, would clarify the mechanisms through which resilience is constructed. Finally, digital ethnographies of online support environments could shed light on how informational and emotional re-sources flow between migrants.

Despite these limitations, we believe that this research is a valuable contribution to critical social psychology and the study of migration, since it sheds light not only on the exclusionary processes that structure Brazilians in Portugal, but also on how they resist, care for each other, and construct forms of collective resilience within adversity. By foregrounding collective resilience, we emphasize that resilience emerges less as an individual trait and more as a collective capacity rooted in shared care practices, solidarity networks, and historical legacies of endurance. By listening to these voices with analytical respect and care, we strive to build knowledge committed to justice, dignity, and inclusion.

## 5. Conclusions

This study highlights how Brazilian immigrants in Portugal face multiple forms of exclusion at work and psychosocial vulnerability. Even so, amid these adversities, forms of care, solidarity, and collective support emerge that operate as essential mechanisms of resilience. More than an individual capacity for resistance, it is the bonds built between migrants, through the exchange of support, listening, and mutual presence, that sustain well-being and permanence.

Regarding policies, the findings suggest different levels of action. In the short term, urgently needed steps are to strengthen mechanisms against discrimination and xenophobia, increase mechanisms for foreign qualification recognition, and institute enforcement of labor laws for the protection of migrant workers. In the longer term, there needs to be structural change, for instance, by adopting a national plan for overall integration, reducing institutional barriers to legalization and nationality, and continued efforts to counter institutional racism and colonial traces that continue to shape labor hierarchies.

By highlighting these everyday practices of support and belonging, this research reinforces the importance of public policies and psychological practices committed to social justice, as well as academic approaches that value the knowledge and strategies produced by migrant populations in precarious contexts.

## Figures and Tables

**Table 1:** Interview script questions.

	Content of the Question
1	Could you tell me about your day-to-day work?
2	How would you describe your current working conditions?
3	How do you assess the impact of the demands of your job on your mental health?
4	Is there someone at work/or a support structure you can lean on in times of difficulty?
5	Have you ever had any difficulty reconciling work with your family/personal issues?
6	Do you believe that any difficulties you’ve had in your workplace may have been aggravated by your nationality?
7	Have you ever noticed any different treatment of yourself or someone else at work because of your nationality?
8	Is there any effort on the part of the workplace to create an inclusive and welcoming environment?

## Data Availability

Due to the confidential nature of the data collected, data are not available for access by those external to the research team.

## Supplementary Materials

The following supporting information can be downloaded at: https://www.mdpi.com/article/10.3390/bs15091283/s1, Table S1: Detailed Sociodemographic Information.
