# Supplementary material for: “We Help Each Other Through It”: Community Support and Labor Experiences Among Brazilian Immigrants in Portugal
Source: Behav Sci (Basel). 2025 Sep 19;15(9):1283. doi: 10.3390/bs15091283 (PMC12467796; doi:10.3390/bs15091283)
Supplement: Supplementary file 1 [file behavsci-15-01283-s001.zip › behavsci-3759805-supplementary.pdf]

Supplementary Table S1. Sociodemographic details 1

	Age	Gender	Sexual Orientation	Relationship Status	Education	Socioeconomic Status	Occupation	Work Duration
1	26	Woman	Bisexual	Single with partner	Up to 12 years of schooling	Low	Coffee shop attendant	6 months
2	31	Man	Gay	Single without partner	Up to 12 years of schooling	Medium	Waiter	6
3	36	Woman	Heterosexual	Married to opposite sex	Doctorate	Medium-High	Assistant Professor	4-5 years
4	37	Woman	Bisexual	Single without partner	Postgraduate/Master	Medium	Accounts Payable Technician	2 years and 7 months
5	32	Man	Heterosexual	Married to opposite sex	Bachelor's degree	Low	Technical Assistant	2 years
6	29	Man	Heterosexual	Single without partner	Technical education	Medium	Production operator	2 years
7	35	Man	Heterosexual	Married to opposite sex	Postgraduate/Master	Medium	Backend Developer	1.5 years
8	28	Woman	Lesbian	Married to same sex	Bachelor's degree	Medium	Advertiser	7 months
9	30	Man	Gay	Single with partner	Postgraduate/Master	Medium	Content Analyst	2 years
10	21	Woman	Lesbian	Single without partner	Up to 12 years of schooling	Low-Medium	Technical course student	6 months
11	36	Woman	Heterosexual	Single without partner	Postgraduate/Master	Medium	Executive Director	2.5 years
12	29	Woman	Lesbian	Married to opposite sex	Bachelor's degree	Low-Medium	Textile Inspector	2 years and 3 months

13	29	Woman	Gay	Married to same sex	Bachelor's degree	Low-Medium	Manual Textile Inspector	2 years and 3 months
14	37	Man	Heterosexual	Single without partner	Bachelor's degree	Medium	Furniture Design	5 years in current company, 10–15 in role
15	38	Woman	Bisexual	Single without partner	Postgraduate/Master	Medium	Medical Translator	6 years
16	37	Man	Gay	Single with partner	Bachelor's degree	Low-Medium	Salesperson	2 years
17	29	Trans Woman	Heterosexual	Dating	Up to 12 years of schooling	Medium	Sex Work	9 years
18	23	Man	Gay	Single with partner	Design technician	Low	Administrative Assistant	1 year
19	31	Trans Woman	Heterosexual	Single with partner	Bachelor's degree	Low	Production Assistant	3 years
20	34	Woman	Lesbian	Married to same sex	Technical education	Medium	Programmer	2 years
21	44	Man	Heterosexual	Married to opposite sex	Bachelor's degree	Medium	IT Solutions Architect	3 years - current company 3 months
22	32	Man	Gay	Single without partner	Postgraduate/Master	Medium	Technology Solutions Coordinator	1 year
23	24	Trans Woman	Pansexual	Single without partner	Vet technician	Medium	OnlyFans	1 year
24	32	Trans Woman	Heterosexual	Single without partner	Up to 12 years of schooling	Low	Club door, waitress	1 year, 2 months
